# A Health App for Evidence-Based Postpartum Information: Development and Validation Study

**DOI:** 10.2196/38706

**Published:** 2023-07-13

**Authors:** Máyra Cármem Silva de Medeiros, Maiara Costa de Oliveira, Leonandro Valério Barbosa Gurgel, Anna Giselle Câmara Dantas Ribeiro Rodrigues, Maria Thereza Albuquerque Barbosa Cabral Micussi, Adriana Gomes Magalhães

**Affiliations:** 1 Faculty of Health Sciences Universidade Federal do Rio Grande do Norte Santa Cruz, RN Brazil; 2 Department of Physiotherapy Universidade Federal do Rio Grande do Norte Natal Brazil; 3 Department of Information Technology Universidade Federal do Rio Grande do Norte Natal Brazil

**Keywords:** women's health, postpartum period, comprehensive health care, health technology, mobile applications

## Abstract

**Background:**

After childbirth, women undergo substantial physical and emotional changes. Therefore, it is important to provide them with information that helps them identify what is expected during this stage, as well as signs and symptoms that indicate complications after they have been discharged from the hospital.

**Objective:**

This study aimed to develop a health app—Towards Motherhood—that provides evidence-based information about the postpartum period and evaluate the usability of the app with the target population.

**Methods:**

This was a validation study involving 80 participants, including 24 professionals from the obstetric health field, 15 professionals from the technology field, and 41 postpartum women. The app was developed using React Native technology. Health professionals evaluated the app’s content using the Content Validity Index, technology professionals completed a validated evaluation to assess the appearance of the app, and postpartum women completed the System Usability Scale (SUS) to measure the usability of the app.

**Results:**

The measurement of content validity using a Likert scale obtained an approval score of 99%. Regarding the app’s appearance, 92% of responses were positive, reflecting favorable approval. The SUS usability score was 86.2, which represents excellent acceptance.

**Conclusions:**

The Towards Motherhood mobile app is a valid tool for promoting self-care during the postpartum period. The app’s evidence-based information, user-friendly design, and high usability make it an essential resource for women during this critical stage of their live.

## Introduction

In the first few days after childbirth, women undergo substantial physical and emotional changes, making it important to provide them with information to identify what to expect during this stage and the signs and symptoms of possible complications after hospital discharge, such as bleeding, pain, and urinary tract infection. Accessible and reliable information, prevention, and care for complications are essential and must be adopted [1,2]

The development of information and communication technology has provided new ways to improve users’ quality of life by monitoring their health status [3]. Telecare, telehealth, and mobile health (mHealth) are components of innovative and frequently used methods [4,5]. These tools can potentially improve people’s health status, are considered tools of great utility for solving or reducing the health problems of individuals or populations, and are user-friendly [6].

It is evident that today, there has been a growth of mobile technologies and apps (mHealth) that contribute to the production of a new modality of health care, in which information regarding people’s health is relevant and universal [7,8].

Given the above, this work aimed to develop a health app—Towards Motherhood—with information about the puerperal phase. Its content was elaborated based on updated scientific knowledge to provide safe content on topics pertinent to this phase. This was then followed by an evaluation on the usability of the app with the target population.

## Methods

### Characterization of Research

This was a validation study conducted at the Januário Cicco Maternity School in the city of Natal, Rio Grande do Norte, Brazil.

### Ethics Approval and Informed Consent

This study was submitted to and approved by the Research Ethics Committee of the University Hospital Onofre Lopes of the Federal University of Rio Grande do Norte (CAAE: 38145320.2.0000.5537). All research participants voluntarily agreed to participate and signed the free and informed consent form.

### Population and Sample

To develop the app, a research group consisting of 2 expert physiotherapists, 3 undergraduate students (one each from IT, physiotherapy, and graphic design), 2 physiotherapy professors, and 1 IT professor held weekly meetings throughout 2021. The app was constructed through database research; group discussions for knowledge translation; evidence-based content preparation and review of screen prototypes; implementation; and subsequent validation of content, functionality, and design.

The evaluation inclusion criteria for health professionals required experience in the obstetric health area and at least a specialist title, whereas the inclusion criteria for IT professionals required previous experience developing mHealth, React Native, or front-end software based on a previous study of mobile app validation. Postpartum women who had given birth at the Januário Cicco Maternity School and were still hospitalized in the institution were included, whereas the exclusion criteria were not agreeing to participate in the research or not signing the free and informed consent form.

### Sample Size

A total of 80 participants were included: 39 professionals (24 health professionals with specialization in obstetrics and 15 IT professionals) and 41 postpartum women. The number of research participants was determined based on articles that use mHealth technology. It has been emphasized that there is no consensus in the international literature on the minimum number of judges, but there is agreement on the importance of clinical experience in the formation of a profile of expertise, as well as the need to balance clinical experience and solid academic training [9-12]. The participants were selected by convenience sampling according to previous research [13].

### Development

**Figure 1:**
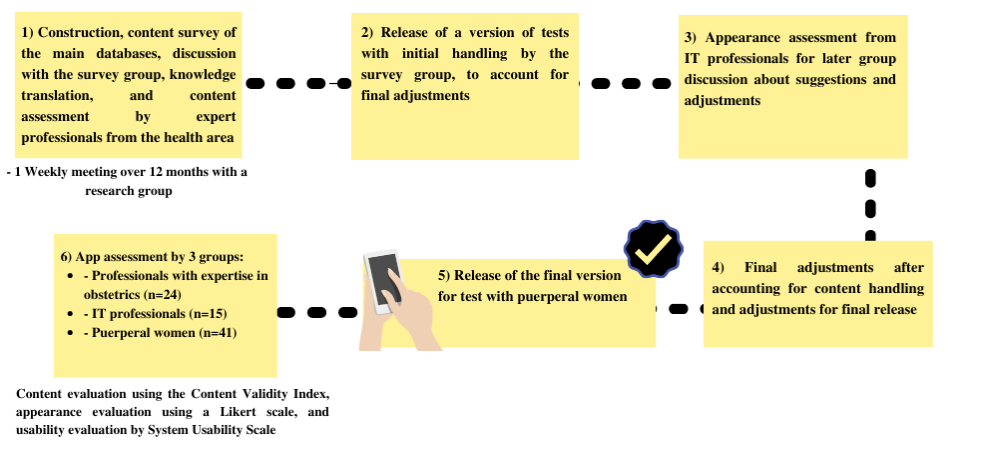
The app development process.

The app was developed using React Native, a JavaScript library created by Facebook (Meta Platforms Inc) to build mobile apps for the Android operating system. The development process consisted of 4 main stages, as illustrated in Figure 1.

### Validation

The expert professionals were contacted via email or telephone and invited to participate in the study. They were provided with information about the research, and upon agreement, they signed the free and informed consent form. The professionals were given access to the app content, evaluated it, and provided feedback.

Postpartum women were approached in the ward, and after signing the free and informed consent form, they were given access to the app through a tablet provided by the evaluator for 20 minutes. After use, participants were requested to complete a form on the Google Forms platform using the tablet.

The process was divided into three stages: (1) evaluation of the content by health care professionals, (2) evaluation of the app’s appearance by IT professionals, and (3) usability assessment with postpartum women.

### Content Assessment

The content of the app was obtained through research in the main databases of Scientific Electronic Library Online, Science Direct, Cochrane, Web of Science, Scopus, and MEDLINE. The following Descriptors in Health Sciences and Medical Subject Headings were used: “Saúde da Mulher” (“Women’s Health” in Portuguese) and “Período Pós-Parto” (“Postpartum Period” in Portuguese).

Documents with up-to-date scientific evidence were selected. The content to be inserted in the app was discussed among the members of the research group, most of whom had experience in the area of women’s health. Subsequently, the information was transcribed into language that was easy for the target audience to understand.

**Figure 2:**
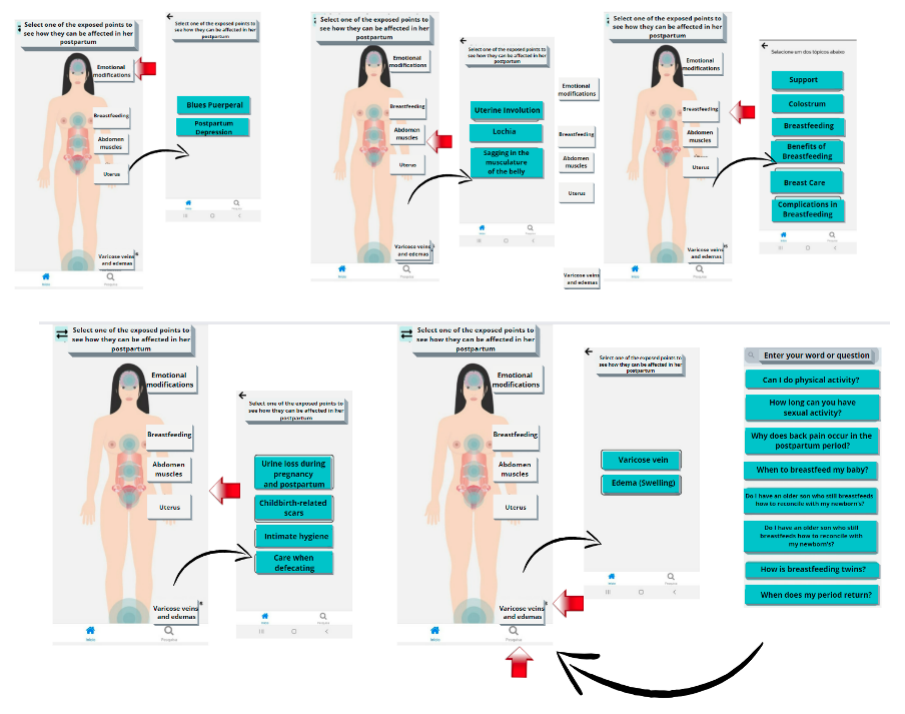
The app content that was evaluated by health professionals with expertise in obstetrics.

The health professionals evaluated the content within the app using a Likert scale with responses ranging from 1 (strongly disagree) to 5 (strongly agree). The evaluated content themes are shown in Figure 2. The Content Validity Index was used to measure agreement on the scores given by specialists for each item, with a final score given as a percentage, which should be greater than 78% [8]. The evaluators with expertise in obstetrics participated in this stage.

### Appearance Assessment

The app’s appearance was evaluated through the User Experience Questionnaire with 6 questions: “Is the language used in the app easy to understand?” “Are the features used in the app implemented correctly?” “Are the features used in the app conducted comprehensively?” “Is the app interface attractive?” “Is the app easy to manage?” and “Does the app provide help in a non-tiring way?” The answers were measured using a Likert scale with 5 possible responses, as in the previous stage. IT professionals participated in this stage, and a minimum of 78% of positive responses was required for approval [6].

### Usability Assessment

Postpartum women evaluated the usability of the application using the System Usability Scale (SUS), a questionnaire that has been translated and validated in Portuguese. It consists of 10 questions, with 5 positively worded statements and 5 negatively worded statements: “I think I would like to use this system frequently,” “I find the system unnecessarily complex,” “I found the system easy to use,” “I think I would need help from a person with technical knowledge to use the system,” “I think the various functions of the system are very well integrated,” “I think the system presents a lot of inconsistency,” “I imagine people will learn how to use this system quickly,” “I found the system clumsy to use,” “I felt confident using the system,” and “I had to learn a lot of new things before I could use the system.” The evaluators responded using a 5-point scale ranging from 1 (strongly disagree) to 5 (strongly agree). The overall score was then calculated on a scale from 0 to 100 points, with a cutoff point of 68 to consider the app as being usable [9,10]

### Statistical Analysis

The sample data were analyzed using the SPSS statistical software (version 20.0; IBM Corp) with a significance level of 5%. The Content Validity Index and User Experience Questionnaire score were calculated by summing up the values of the answers and presenting them as a percentage. The SUS score was calculated as follows: for odd items, 1 was subtracted from the position on the scale, and for even items, 5 was subtracted from the position on the scale; then, all items were summed and multiplied by 2.5 to obtain the overall usability score of the system.

## Results

For the development of the app, an integrative review was conducted in the main databases of Scientific Electronic Library Online, Science Direct, Cochrane, Web of Science, Scopus, and MEDLINE.

Initially, the app was named “Towards Motherhood” and was designed for offline use with free access on the Android platform. The app’s main menu offers 5 topics for exploration: Emotional Changes, Breastfeeding, Abdominal and Uterine Muscles, Varicose Veins, and Edema, as shown in Figure 2. Each topic includes subtopics for easy navigation and access to information.

The content validation process included 24 health professionals with expertise in obstetrics, of which 83% (n=20) were women. The group included 33% (n=8) physiotherapists, 29% (n=7) medical professionals, and 38% (n=9) nurses. More than half (n=13, 54%) had a specialization degree and experience in the public health system, 25% (n=8) had a master’s degree, 8% (n=2) had a doctorate degree, and the remaining 13% (n=3) were undergraduate students. Table 1 displays the answers and comments provided by the health professionals, with the suggestions discussed by the research group and accepted based on pertinence and scientific evidence. Breastfeeding was the topic with the highest number of suggestions, whereas the content on the postpartum period and its stages had a 100% agreement and no suggestions. Content validation was conducted through the Likert scale, obtaining a score of 97%.

The appearance of the app was evaluated by 15 IT professionals, comprising of 67% (n=10) male and 33% (n=5) female participants. They commented that the app was easy to use and had good understanding of the functionalities of the elements. Whenever there was a disagreement among the specialists on any item of the app, they proposed a new statement or new title for the menu, recommended the inclusion of additional information, or suggested the inclusion of a new item. The approval rate was 92% positive responses, which was favorable. The responses are presented in Table 2.

**Table 1:** Answers and comments from obstetric health professionals (n=24).

Subject	Strongly disagree, n (%)	Partially disagree, n (%)	Neutral, n (%)	Partially agree, n (%)	Strongly agree, n (%)	Suggestions	Analysis
Puerperal blues	0 (0)	0 (0)	0 (0)	3 (13)	21 (87)	Emphasize that the time to rest and sleep is essential, and the support network is essential to take care of the baby in these moments	Accepted
Postpartum depression	0 (0)	0 (0)	0 (0)	8 (33)	16 (67)	Seek professional help	Accepted
Breastfeeding	0 (0)	0 (0)	0 (0)	7 (29)	17 (71)	Create a topic on how to make the correct handle	Accepted
Uterine involution	0 (0)	0 (0)	0 (0)	4 (17)	20 (83)	Seek medical attention if the pain is not ceasing	Accepted
Abdominal diastasis	0 (0)	0 (0)	0 (0)	6 (25)	18 (75)	Show images of some movements used to minimize postpartum diastasis	Evaluation required
Urinary incontinence	0 (0)	0 (0)	0 (0)	4 (17)	20 (83)	The term “postpartum incontinence of urine” looks like a classification	Accepted
Scars arising from childbirth	0 (0)	1 (4)	0 (0)	4 (17)	19 (79)	Hygiene practices	Accepted
Lochia	0 (0)	0 (0)	0 (0)	8 (33)	16 (67)	Details about duration and warning signs	Accepted
Intimate hygiene	0 (0)	0 (0)	0 (0)	2 (8)	22 (92)	Avoid tampons	Accepted
Care in defecation	0 (0)	0 (0)	0 (0)	2 (8)	22 (92)	Better explanation about the position of squats	Accepted
Varicose vein	0 (0)	0 (0)	0 (0)	3 (13)	12 (87)	Orientation about the importance of talking with the doctor about compression stockings	Accepted, with physiotherapists added to the suggestion
Edema	0 (0)	0 (0)	0 (0)	3 (13)	12 (87)	Orientation about water intake	Accepted
Healthy habits	0 (0)	0 (0)	0 (0)	4 (17)	20 (83)	Well-being and leisure	Accepted
Postpartum sexual activity	0 (0)	0 (0)	1 (4)	3 (13)	20 (83)	Importance of talking about contraception	Accepted
Back pain in postpartum period	0 (0)	0 (0)	1 (4)	0 (0)	23 (96)	Exercise videos	Evaluation and prescription of exercises should be performed individually

**Table 2:** Responses of IT professionals on the app’s appearance (n=15).

	Strongly disagree, n (%)	Partially disagree, n (%)	Neutral, n (%)	Partially agree, n (%)	Strongly agree, n (%)
Is the language used in the app easy to understand?	0 (0)	0 (0)	1 (7)	4 (27)	10 (67)
Are the features used in the app implemented correctly?	0 (0)	0 (0)	0 (0)	7 (47)	8 (53)
Are the features used in the app conducted comprehensively?	0 (0)	0 (0)	0 (0)	5 (33)	10 (67)
Is the app interface attractive?	0 (0)	2 (13)	3 (20)	5 (33)	5 (33)
Is the app easy to manage?	0 (0)	0 (0)	1 (7%)	3 (20)	11 (73)
Does the app provide help in a non-tiring way?	0 (0)	0 (0)	0 (0)	5 (33)	10 (67)

The usability of the application was assessed by 41 postpartum women aged 18 to 40 years, with the majority (n=27, 66%) having completed high school education, followed by 19% (n=8) who were literate and 15% (n=6) with higher education. The majority (n=25, 61%) of the participants were single and the rest (n=16, 39%) were in a stable union. The users’ feedback is presented in Table 3, where their comments on the Google Forms questionnaire included “I found it very informative,” “Good and easy to use,” “I enjoyed the experience,” “The information was very useful,” and “Very good.” Usability was evaluated using the SUS, which yielded a score of 86.2, indicating excellent acceptance. However, the available version is a prototype developed for app validation testing and is not yet available for free access.

**Table 3:** User responses on the usability of the app (n=41).

	Strongly disagree, n (%)	Partially disagree, n (%)	Neutral, n (%)	Partially agree, n (%)	Strongly agree, n (%)
I think I’d like to use this system often	1 (2)	3 (7)	1 (2)	11 (27)	25 (61)
I find the system unnecessarily complex	30 (73)	3 (7)	3 (7)	3 (7)	2 (5)
I found the system easy to use	3 (7)	1 (2)	0 (0)	5 (12)	32 (78)
I think I would need help from a person with technical knowledge to use the system	24 (59)	3 (7)	0 (0)	10 (24)	4 (10)
The various functions of the system are very well integrated	0 (0)	0 (0)	0 (0)	5 (12)	36 (88)
The system presents a lot of inconsistency	34 (83)	3 (7)	1 (2)	1 (2)	2 (5)
I found the system clumsy to use	38 (93)	1 (2)	0 (0)	1 (2)	1 (2)
I felt confident using the system	1 (2)	1 (2)	1 (2)	3 (7)	35 (85)
I had to learn several new things before I could use the system	23 (56)	8 (20)	0 (0)	5 (12)	5 (12)

## Discussion

### Principal Findings

Developing a technology to facilitate the acquisition of evidence-based content for a stage of life that brings countless doubts to women is of utmost relevance. This fact can be supported by the identification that such technologies are scarcely available in the main web stores and are not widely published in the major health journals [14].

The main objective of this study was not only to create an app about postpartum care but also to develop a technology that aligns with self-care for women, as empowering these women is essential to avoiding complications in the postpartum phase. As a refinement of this app, health professionals with extensive experience in obstetrics were able to give their opinion on the content, as well as find the necessary areas of improvement using their practical experience to determine what they perceive to be the main difficulties and doubts of puerperal women. Existing work in this area remains incomplete, with a limited sample size and a need of further investigation [15].

Knowledge translation is a means to communicate scientific evidence in an effortless way, with the objective of being effectively understood and applied in real life and influencing the creation of new products and technologies. In the context of education and health promotion, care should be taken regarding the adequacy of the language used to facilitate understanding. Popularly used words should preferably be used, and technical terms should be restricted to what is strictly necessary [12,16].

Learning is directly influenced by the social and cultural beliefs of the environment in which it is embedded, and therefore, the content of the app was selected with the aim of not disrespecting these issues. As a refinement, an attempt was made to adapt the guidance to all audiences without losing its scientific nature, which is supported by relevant literature. The content is presented not only in text but also in images to facilitate understanding.

According to literature, a SUS score above 68 indicates acceptable usability, whereas a score of 85 or above is related to excellent approval of software or applications. The mean SUS score for the Towards Motherhood app reached these parameters, as seen in a broad examination of the SUS [15,17].

Analysis of the SUS items showed greater variance in responses for “I think I would need help from a person with technical knowledge to use the system” and “I felt confident when using the system.” This result could be attributed to the low level of education of users, highlighting the need for simplified language, more images, and a dynamic app with less text. In terms of the appearance of the app, most technology professionals found it easy to manage and helpful but only partially agreed on its coverage and attractiveness. These points will be prioritized in future updates.

The Family Health Strategy is an ideal scenario for promoting the use of this tool since the health professionals in these teams aim to expand patient self-care and promote the accountability of care for the user [18]. The app also reinforces the information given by the multidisciplinary team in the hospital, as many women may be tired or focused on their newborn during postpartum visits and did not absorb the orientations well.

The positive results related to usability and potential for app use motivate future updates to improve functionality, update content, and add new features.

### Limitations

Audio and video content was suggested to be included in the app, but this would cause the app to not be compact and it would move away from the research proposal.

### Conclusion

The Towards Motherhood mobile app is deemed to be a valid tool to promote self-care. Through the search in web stores and a literature review, no other app with a similar objective was found. In future updates, additional functions can be integrated into the app, and it can be translated into other languages to cater to a wider range of populations. A summary of this study is presented in Textbox 1.

**Summary table.**

**What was known about the subject?**

• The use of health apps is a growing trend worldwide and is seen as an attractive and facilitating option.

**What did this study add to our knowledge?**

• This study highlighted the need for postpartum apps as they are currently scarce in web stores, which only offer them for the pregnancy period.

• This study demonstrated the importance of knowledge translation, providing scientific and reliable content in digital environments.

• The development of a multiprofessional technology with a broad vision was also emphasized.

## Abbreviations

mHealth: mobile health
SUS: System Usability Scale
